# Human factors and airway management: focus on the Swiss Airway Guidelines

**DOI:** 10.3389/fmed.2026.1901921

**Published:** 2026-08-28

**Authors:** Christian Balmer, Gabriele Casso, Patrick Schoettker, Georges Louis Savoldelli

**Affiliations:** 1 Department of Anaesthesia, Valais Hospital, Sion, Switzerland; 2 Division of Anaesthesiology, Department of Acute Care Medicine, Geneva University Hospitals, Geneva, Switzerland; 3 Clinic of Cardiovascular and Thoracic Anaesthesia and Intensive Care, Istituto Cardiocentro Ticino EOC, Lugano, Switzerland; 4 Department of Anaesthesiology, Lausanne University Hospital and University of Lausanne, Lausanne, Switzerland; 5 Department of Anaesthesiology, Pharmacology, Intensive Care and Emergency Medicine, Faculty of Medicine, University of Geneva, Geneva, Switzerland

**Keywords:** adaptive complex systems, airway management, decision-making, ergonomics, human factors, patient safety, SEIPS model

## Abstract

Although decision-making in airway management is crucial for performance and outcomes, it often deteriorates in crisis situations. Studies suggest that human decision-making can be unreliable in such situations and must be supported to reduce reliance on exceptional human performance to ensure safety. Airway guidelines provide essential evidence-based information intended to maximize the likelihood of successful airway management. However, the linear, textual and inflexible design of most algorithms does not align with the naturalistic decision-making processes involved in clinical activity. They fail to consider how decisions are made under time pressure. A user-centered design that considers the cognitive challenges of high-stakes, time-pressured airway situations might be more effective in supporting decisions at the point-of-care. The science of human factors and ergonomics has become central to airway management, particularly in supporting human performance in highly demanding cognitive environments. It provides key strategies to support cognitive load, situational awareness, and ergonomic design, maximizing the advantages of new technologies by integrating them into workflows and promoting their intuitive use in stressful situations. Consequently, human factors and ergonomics might effectively promote collaborative decision-making at the sharp end. Guided by principles of human factors, the Fondation Latine des Voies Aériennes designed the Swiss Airway Guidelines, endorsed by the Swiss Society of Anesthesiology and Perioperative Medicine to provide clinicians with point of care guidance and support during airway management. They combine color- and icon-coded cognitive aids including key mnemonics aligned with an ergonomic airway cart. They also encompass a multimodal educational program and an implementation strategy. This system-based intervention aims to optimize decision-making in high-pressure, front-line context. By recognizing the cognitive capabilities and constraints of operators in crisis situations, it employs a holistic design approach to deliver comprehensive decision support. This narrative review uses the lens of human factors and ergonomics to describe the Swiss Airway Guidelines, drawing on the Systems Engineering Initiative for Patient Safety 3.0 and 101 frameworks. Our aim is to emphasize its importance in successfully managing airways, and to illustrate that reliable airway safety stems from systems designed to support human performance.

## 1 Background

Airway management in crisis situations remains associated with suboptimal performance and decision-making, despite decades of technical innovation and guideline development (1). Time pressure, stress, and uncertainty, frequently trigger maladaptive behaviors such as rushing (“hurry-up syndrome”), fixation on technical issues, reduced communication, and loss of situational awareness (SA) (2). Even experienced clinicians are vulnerable, as cognitive limitations inherent to human decision-making can lead to errors under high workload conditions (2).

Substantial efforts have been made to enhance airway safety through national guidelines, improved individual technical training, team-based training using simulation and various technological advances such as second-generation supraglottic devices, video-assisted laryngoscopy, peri-intubation oxygenation strategies, and point-of-care ultrasound (3). Nevertheless major airway-related complications (including death, brain injury, emergency surgical airway, or unplanned critical care admission) still persist (4). These adverse events continue to represent a significant proportion of anesthesiology malpractice claims (5). Growing evidence suggests that failures in social and cognitive skills (non-technical skills), such as leadership, teamwork, communication, cognitive load management, and SA, are key contributors to airway-related complications (4, 6–8). The delayed transition to rescue strategies and the lack of availability or improper use of new technologies also contribute to preventable morbidity and mortality in airway management. Airway crises are therefore not purely technical failures but systemic breakdowns occurring within complex sociotechnical environments (9, 10).

Despite recognition that airway management is embedded within a dynamic sociotechnical system, most safety interventions continue to focus predominantly on technical performance and normative guideline adherence (“work-as-prescribed”), rather than on how decisions are actually made under real-world constraints (“work-as-done”) (11). Furthermore, airway guidelines cannot fully address every nuance of individual anatomy, physiology, patient-specific factors and context (12). Decision-making in high-stakes airway situations largely follows recognition-primed processes, whereby clinicians rapidly match situational cues to prior experiences and generate actions accordingly (2, 11, 13). However, existing airway guidelines are typically linear, text-based, and cognitively demanding. Their design does not adequately align with naturalistic decision-making under time pressure, uncertainty, and high cognitive load (11, 14–16).

Lastly, insufficient attention has been paid to integrating Human Factors and Ergonomics (HFE) principles into airway system design (1, 3). Without deliberate attention to cognitive load, SA, ergonomic interfaces, and shared mental models, even well-designed technologies and evidence-based guidelines may fail at the point of care (3). A real user-centered, systems-oriented approach to airway management still remains underdeveloped in current practice (1, 11, 17).

To address this gap, we adopted a HFE approach, drawing on the Systems Engineering Initiative for Patient Safety (SEIPS) 3.0 (18) and SEIPS 101 (19) frameworks, to reframe the theoretical foundations of the previously published Swiss Airway Guidelines (SWAGs) (10) developed by the Fondation Latine des Voies Aériennes (FLAVA). FLAVA is a not-for-profit organization recognized as the airway interest group of the Swiss Society of Anaesthesia and Perioperative Medicine (10, 20).

While the original design and rationale of the SWAGs were described in detail in our foundational publication (10), the present article extends this work in three specific ways: (1) it reframes the SWAGs’ development and clinical implementation through the lens of Human Factors and Ergonomics, using the SEIPS 3.0 and 101 models to analyze the work system components (tools, tasks, organization, person, environment) that shape airway management performance, an analytical framework not applied in the foundational article (10); (2) it introduces our local implementation strategy, framed as a system-level approach rather than a single-artifact intervention; and (3) it introduces and describes the airway bag, a subsequent development building on the SWAGs concept, which has not been previously reported.

We suggest how integrating HFE principles into airway system design can reduce reliance on individual performance and improve safety in complex clinical settings.

## 2 Approach/methods

This article is presented as a narrative review. Relevant literature was identified through searches of PubMed, Embase, and Google Scholar using combinations of the terms *airway management*, *cognitive aids*, *checklists*, *situational awareness*, *human factors*, *ergonomics*, *decision-making*, and *crisis resource management*. Additional references were identified through citation tracking of key publications and consensus guidelines. Studies were selected for their relevance to the design and implementation of cognitive aids in airway management and related high-risk clinical environments. The design principles proposed in this review were derived through an iterative synthesis of the published evidence, complemented by the authors’ clinical expertise in airway management and human factors.

## 3 Human factors and ergonomics principles to design the SWAGs

Improving patient safety requires a greater emphasis and a better integration of human factors in existing guidelines, to put it simply we need to shift from reliance on the “heroic” individual clinician toward safer system-based approaches (21).

HFE are central to airway management in cognitively demanding environments, providing strategies to manage cognitive load, enhance SA, and optimize ergonomic design (3). These approaches facilitate the effective integration and intuitive use of technologies under stress and support collaborative decision-making at the point of care (2, 3). Understanding task complexity and human decision-making limitations can inform the design of decision-support tools (11), with system-level interventions proving more effective than attempts to modify individual behavior (22). Accordingly, tools and technologies should be designed to support expert teams by enhancing cognitive performance and reliability (11).

The SWAGs propose a holistic approach that aligns all components of the work system to optimize implementation, improve patient safety, and support user wellbeing. They integrate color- and icon-coded cognitive aids, structured mnemonics aligned with an ergonomically organized airway cart, and a multimodal implementation strategy. Rather than replacing international guidelines, they are designed to complement them by supporting clinicians in both anticipated and unanticipated airway difficulties.

The proposed benefits remain theoretical until confirmed through prospective evaluation. The SWAGs should be regarded as a hypothesis-informed design framework. Their true value will depend not only on sound design principles but also on successful implementation and prospective evaluation in clinical practice or simulation.

## 4 SEIPS frameworks and the SWAGs model description

**FIGURE 1:**
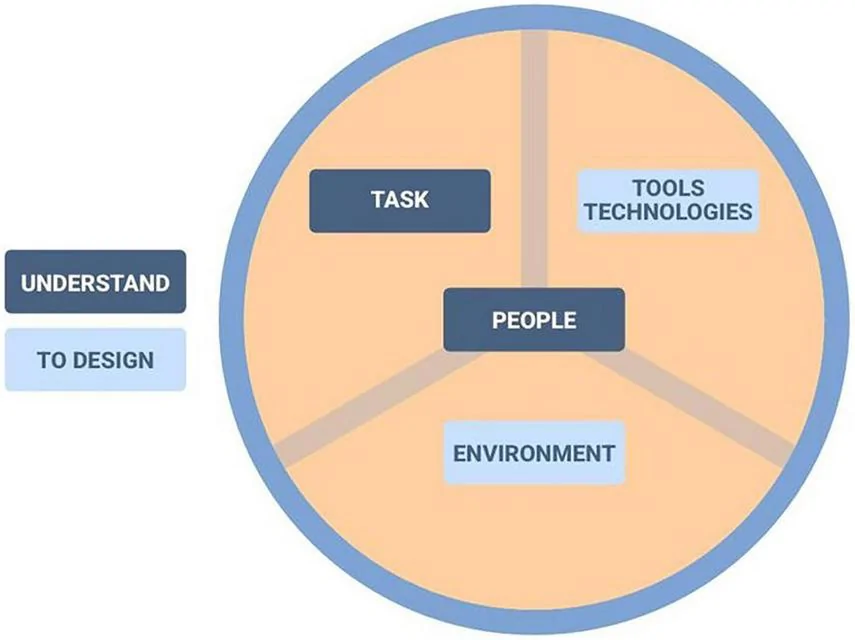
SWAGs user-centered approach, inspired by the work of Carayon et al. (18, 19), underpins its human factors framework.

SEIPS 101 (19) defines the structural components of healthcare systems, while SEIPS 3.0 (18) extends this by framing them as dynamic, adaptive sociotechnical systems evolving over care trajectories. Building on both frameworks, we describe SWAGs from a human factors perspective focused on interactions within the work system. This user-centered approach (Figure 1) starts with analyzing user and task demands, then informs the design of tools, technologies, and environments to improve performance while reducing workload, stress, and preventable harm.

### 4.1 Understanding task and people

#### 4.1.1 Understanding task

Although airway algorithms provide an essential framework for action, they incompletely capture the dynamic nature of clinical practice (15, 23). Airway management can be conceptualized as a complex adaptive system (CAS) (24, 25) operating within a volatile, uncertain, complex, and ambiguous (VUCA) clinical environment (26), where safe and effective performance depends on the adaptive capacity of clinicians and teams to respond to evolving conditions (Resilience engineering) (27, 28) (Table 1).

**TABLE 1:** HFE perspective on airway management.

Framework	Focus on airway management	Question addressed in airway management
VUCA	Operational context	Why is environment challenging?
Complex adaptive system	Nature of the system	How does the system behave under changing conditions?
Resilience engineering	Mechanism of adaptive performance	How is performance maintained despite variability?

This perspective aligns with contemporary human factors and patient safety theories, which view safety as emerging from the adaptive capacity of clinicians and teams to manage complexity and variability, rather than from procedural compliance alone (18, 28–31).

##### 4.1.1.1 VUCA as the operational context of airway management

Airway management is performed in a VUCA environment (26). Volatility is reflected in the potential for rapid physiological deterioration, whereby oxygen desaturation, hemodynamic instability, or airway obstruction may abruptly transform a controlled procedure into a life-threatening emergency. Uncertainty arises from the limited ability to predict airway difficulty (32) and from continuously evolving clinical conditions requiring ongoing reassessment. Complexity emerges from interactions between anatomical, physiological, technical, cognitive, and organizational factors, while ambiguity stems from decision-making under incomplete or conflicting information and competing clinical priorities.

These conditions place substantial demands on human performance. Time pressure, cognitive workload, information overload, and rapidly changing clinical states challenge situational awareness, decision-making, communication, and coordination (33, 34). Consequently, successful airway management depends not only on technical expertise but also on the ability of clinicians and teams to maintain shared understanding, anticipate evolving risks, and adapt their actions under pressure (10).

##### 4.1.1.2 CAS as the intrinsic nature of airway management

Airway management can be conceptualized as a CAS (25, 35) in which outcomes emerge from continuous interactions among clinicians, patients, technologies, monitoring systems, and organizational factors. These interactions are dynamic, nonlinear, and highly sensitive to context, such that small physiological or operational perturbations may lead to disproportionate consequences.

From a human factor’s perspective, performance is not solely determined by individual competence or adherence to protocols. Rather, it emerges from the capacity of clinicians and teams to coordinate actions, share information, manage workload, and adapt to changing conditions. Safety therefore cannot be understood exclusively as the prevention of error, but as an emergent property of effective system functioning, arising from interactions between people, technology, and the clinical environment (36, 37).

##### 4.1.1.3 Resilience engineering as the mechanism of successful performance

Viewing airway management as a CAS operating within a VUCA environment provides a useful framework for resilience engineering and Safety-II (30). Within this perspective, safety is understood as the ability of clinicians and teams to adapt successfully to variability, uncertainty, and unexpected events to maintain effective performance.

Resilience is expressed through processes such as anticipation, monitoring, adaptation, and learning. During airway management, clinicians continuously adjust strategies in response to physiological changes, procedural feedback, equipment limitations, and evolving team dynamics. These adaptive behaviors support the maintenance of situational awareness, effective communication, and coordinated action despite adverse conditions.

Taken together, these perspectives have important implications for airway management, education, and patient safety. They challenge purely linear approaches to airway crises and emphasize that safe performance relies on adaptive decision-making, cognitive flexibility, communication, and team coordination. This supports both user-centered decision support design and training strategies grounded in simulation, crisis resource management, and human factors, highlighting the central role of adaptive capacity in managing airway care under uncertainty and complexity.

#### 4.1.2 Understanding people

Building on the conceptualization of airway management as a complex adaptive task operating in a VUCA environment, resilience engineering shifts the focus toward human performance as the primary determinant of system functioning. It posits that successful care delivery depends on clinicians’ ability to adapt to contextual variability, manage system constraints, and balance competing risks, costs, and benefits (25, 35). Human resilience is therefore understood as the capacity to continuously adjust care processes in response to patient-specific conditions and clinical uncertainty in order to achieve desired outcomes (38). In this view, clinicians constitute the principal source of system resilience in airway management. As clinical outcomes are shaped by real-world decision-making under uncertainty, guideline development should be informed by how practitioners actually make decisions in practice (2, 3, 14, 15, 37).

**FIGURE 2:**
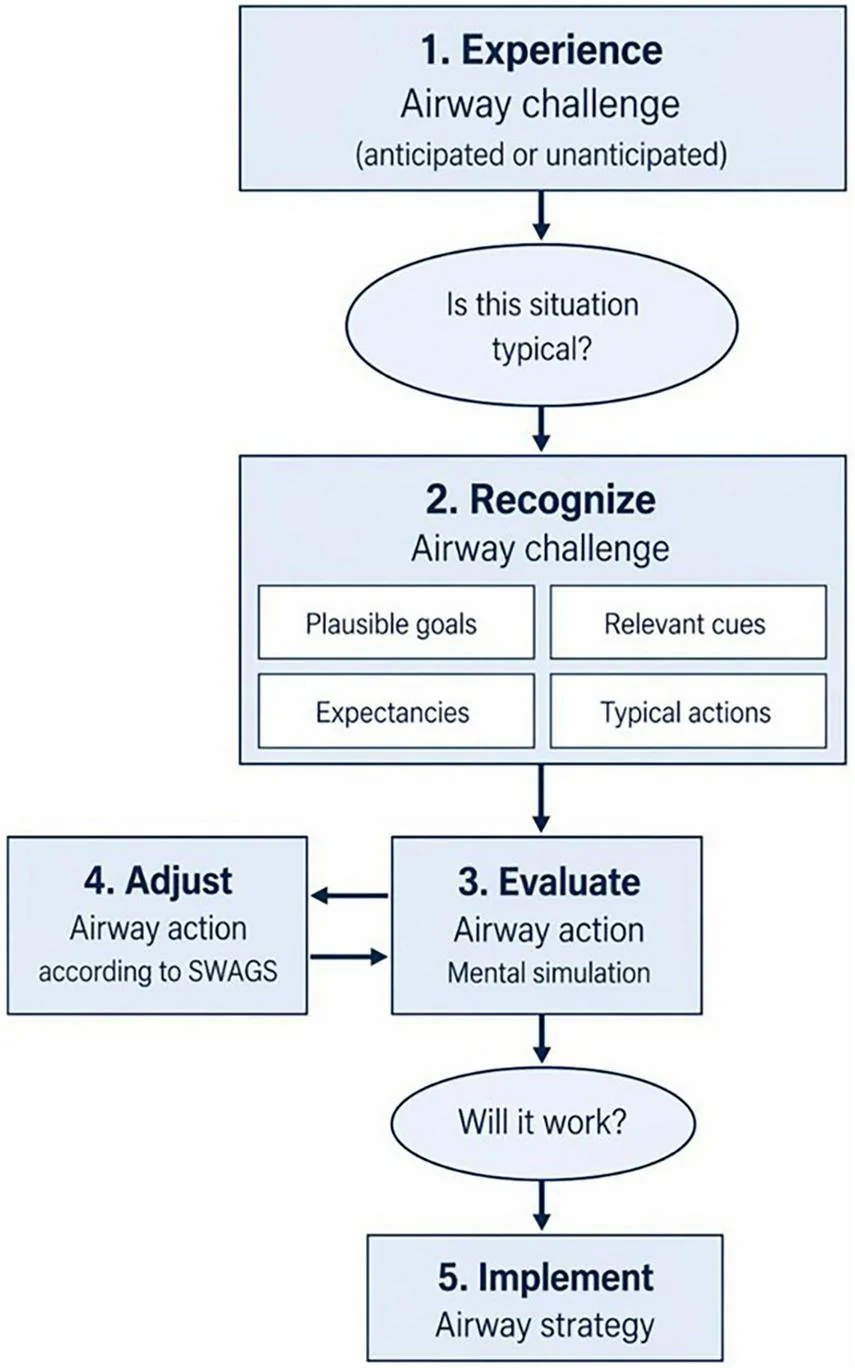
RPD model, inspired from Klein (40) and adapted to the airway management context.

With respect to decision-making, humans demonstrate a high level of proficiency in detecting subtle deviations from routine workflow and rapidly interpreting complex situations through pattern recognition (38). This process involves matching the current situation to previously acquired experiential knowledge. Notably, even a partial correspondence between a present scenario and an existing mental model may be sufficient to generate a viable course of action, a mechanism referred to as pattern matching (39). This cognitive process is typically rapid, requires minimal conscious analytical effort, and frequently yields satisfactory outcomes. It is formally described as Recognition-Primed Decision-making (RPD) (40), which constitutes the predominant model within the broader framework of Naturalistic Decision-Making (NDM) (11). One small ergonomics qualitative study suggested that, during airway management, experienced clinicians often rely on rapid pattern recognition for most airway management decisions (11). In RPD (Figure 2), individuals rapidly interpret situations by matching them to patterns stored in long-term memory, which shape expectations, relevant cues, goals, and typical actions. Potential actions are mentally simulated and, if deemed appropriate, implemented. This process is fast, largely unconscious, and cognitively efficient for experienced practitioners, often described as intuitive (39, 41).

Within the NDM framework, SA at the individual or team level constitutes a fundamental prerequisite for effective decision-making and action (13). SA operates as a central driver of the decision-making process, shaping perception, interpretation, and subsequent behavioral responses (13). Accurate SA requires the mobilization of cognitive resources, particularly attention and memory, both of which are inherently limited in human information processing (33, 42, 43). Among these resources, working memory, responsible for the temporary storage and manipulation of task-relevant information, is especially vulnerable to overload. Excessive informational input and elevated stress levels can saturate working memory capacity, thereby constraining the brain’s ability to integrate and process information efficiently (6, 44). Such overload increases the likelihood of cognitive errors and suboptimal decisions.

The cognitive load refers to the amount of effort and information processing required by our working memory at any given moment. Intrinsic load is linked to the task itself (e.g., delivering oxygen to the lungs) and is hardly modifiable. Extraneous load arises from the way information is presented and is therefore highly influenced by the design of the supporting tool or cognitive aid. Germane load, although not explicitly addressed in this discussion, is linked to the processing that facilitates the integration of information into long-term memory. A comprehensive description of cognitive load theory is beyond the scope of this article; further information can be found in the literature (45, 46).

Cognitive overload leads to unproductive behaviors such as rushing (the “hurry-up syndrome”) (47) and inappropriate responses (e.g., focusing on a technical problem, i.e., the “tunnel effect”) (43) all of which can impact the quality of SA. Although estimates vary according to methodology and clinical context, analyses from high-risk clinical settings have suggested that situational awareness failures contribute to a large proportion of critical incidents, with one study of 200 anesthesia and critical care incident reports reporting a frequency as high as 81.5% (48).

Understanding the task and people guided the design of the SWAGs. They target four elements to enhance human capabilities and mitigate limitations in high-pressure airway management situations:

1. Promoting RPD by providing goal-oriented information displays and making critical cues related to key features of schemata salient in the interface design.
2. Promoting a shared mental model (sMM) by fostering dynamic SA.
3. Reducing the cognitive load on the operator to make it simple to do the right thing.
4. Aligning the dynamic nature of airway management to the design of decision support by opting for a circular shape.

**FIGURE 3:**
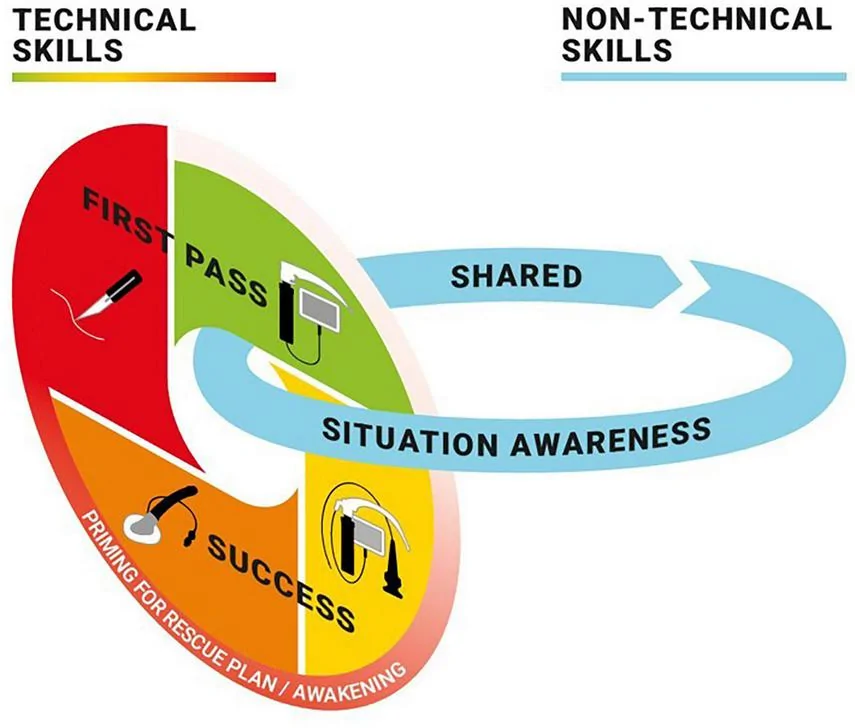
Objectives aimed at technical and non-technical circles. Technical circle (color-coded circle): Salient, action-oriented cues support intuitive decision-making and technology use under stress, intended to improve first-pass success. Non-technical circle (blue circle): Structured huddles enhance shared situational awareness, aiming at supporting team decision-making and coordination.

In light of the potential intended benefits of optimizing first-pass success and promoting the development of a shared mental model (see Figure 3), the SWAGs aim to enhance performance and patient safety in airway management, while mitigating adverse outcomes, including operator stress and preventable patient harm.

### 4.2 Designing tools and technologies

As Lomax et al. state: “The reliability, availability, functionality and usability of technologies and tools affects clinical work and outcomes by promoting or reducing opportunities for errors, enhancing performance, enabling better awareness and decision-making, or being able to work faster” (49).

Different decision pathways call for different decision supports and should be suitable for the specific environment they are intended to be used in (11). Although international algorithms provide an essential foundation for education and training, their predominantly textual format limits their effectiveness during time-critical clinical activities (50, 51). In contrast, cognitive aids function as “implementation tools” intended for real-time use during task execution (50, 51), distinguishing them from traditional protocols or standard operating procedures (14).

Cognitive aids are designed to guide users in a manner that minimizes errors and omissions while enhancing the speed, fluency, and reliability of performance (14). A systematic review reported an average reduction in decision time of approximately 45 s (52), an advantage of particular clinical relevance in time-sensitive procedures such as airway management, although the included studies were heterogeneous and the magnitude of benefit may vary by context. Importantly, these tools are intended to support trained expert teams in maintaining high levels of performance, rather than to compensate for deficits in novice practitioners facing situations beyond their expertise (51).

Beyond improving critical airway management skills, cognitive aids have been shown to enhance overall performance and increase provider confidence (52). They also reduce reliance on working memory and attentional resources (3), thereby mitigating cognitive load and supporting more consistent task execution. As such, they represent an effective form of decision support in high-acuity settings.

To facilitate NDM, where salient cues constitute key components of mental models, and to promote SA (53) interface design should emphasize goal-directed information displays and highlight critical cues. Consistent with the “work-as-done” perspective, this pragmatic focus on “what to do” is likely to be more efficient in practice than comprehensive “how-to” guidance centered on adherence to local policies (51, 54). The development of cognitive aids should follow an iterative refinement process informed by user feedback, ensuring adaptability to evolving clinical environments (14).

However, suboptimal design, particularly when tools are poorly adapted to their intended context, along with inappropriate content not grounded in best practices or misalignment with existing training frameworks may inadvertently promote incorrect action sequences and result in harmful outcomes (14).

#### 4.2.1 The SWAGs cognitive aids

**FIGURE 4:**
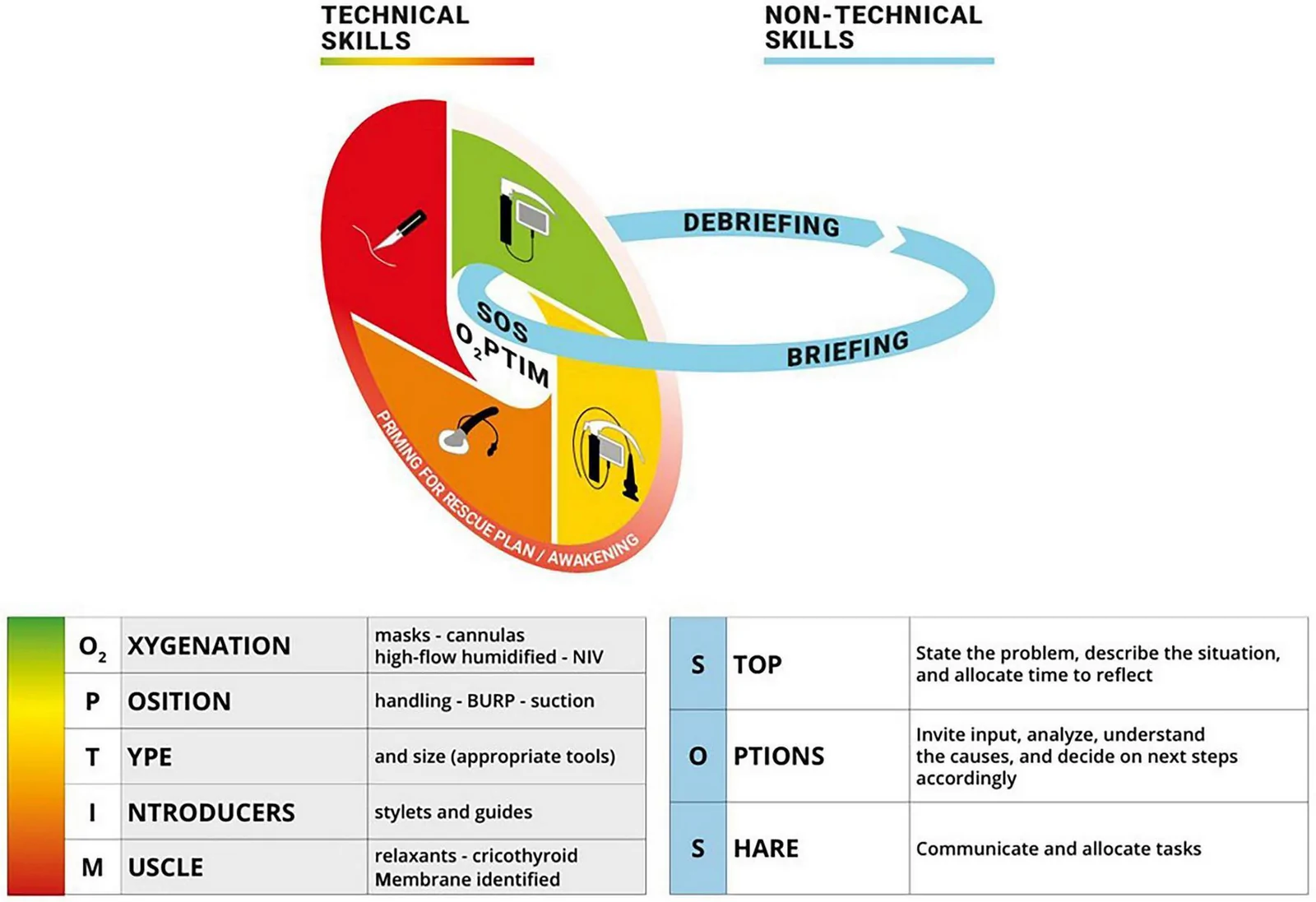
Overview of the SWAGs cognitive aids.

The SWAGs cognitive aid includes two circular figures and two mnemonics (Figure 4): the technical and the non-technical circles and the mnemonics O_2_PTIM and SOS. Simplicity and the intuitive color-icon visual are intended to decrease cognitive load, foster SA and prompt clinicians to take action.

#### 4.2.2 The technical circle and its mnemonic

**FIGURE 5:**
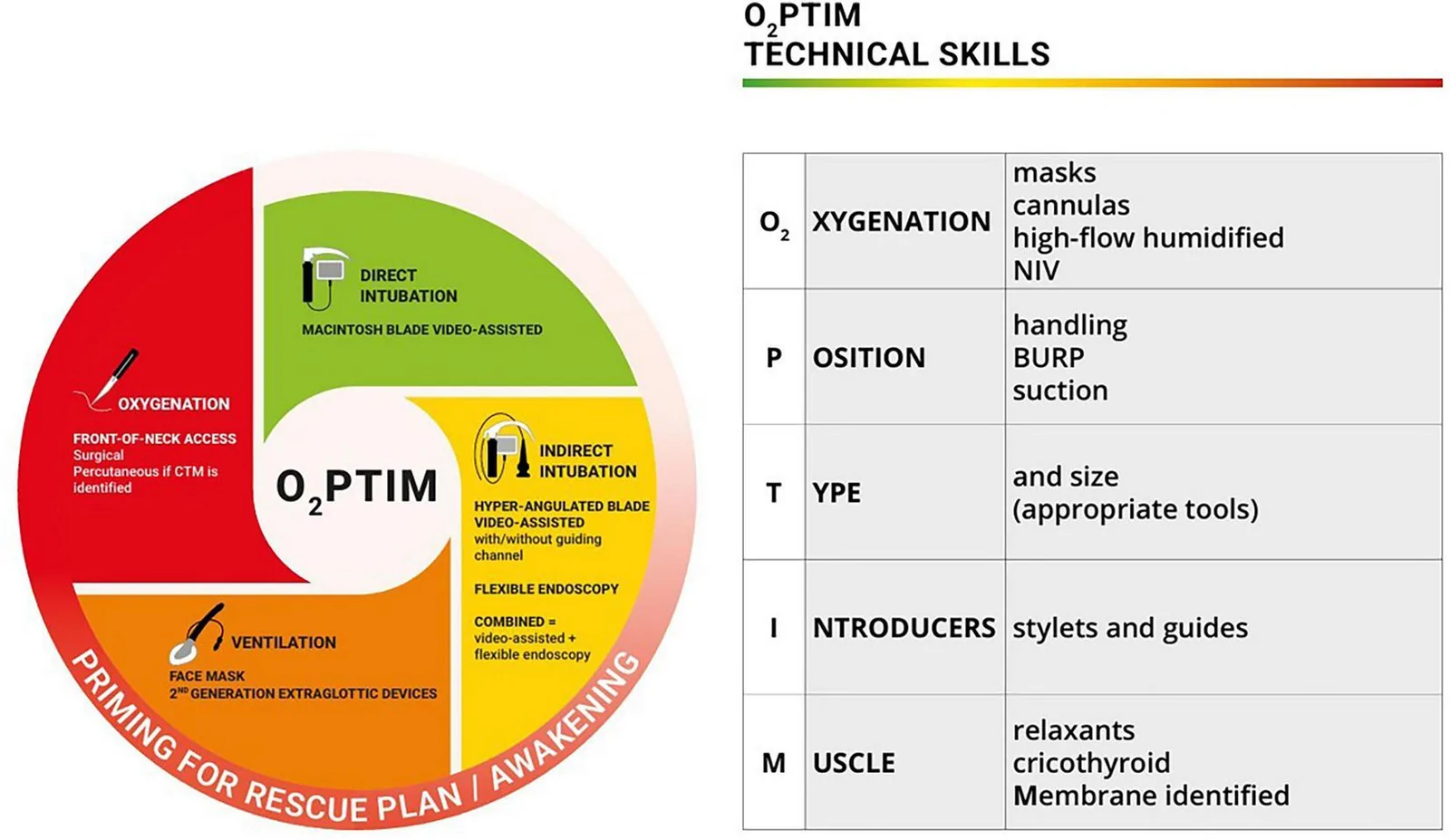
Overview of the SWAGs technical cognitive aids. A full description can be found elsewhere (10).

The technical circle, along with its associated mnemonic, is designed to facilitate NDM by providing critical, goal-directed cues pertaining to the essential features of schemata. These cues are highlighted using intuitive color-coded visuals. The primary purpose of the technical circle is to prompt operators to take timely actions, to ensure a seamless transition between airway management plans, and to serve as an effective safeguard against the omission of a rescue strategy (see Figure 5).

Furthermore, this design supports SA in alignment with SA-oriented design principles (53) and reduces cognitive load by presenting information in an organized manner that corresponds with the layout of the SWAGs airway cart.

**FIGURE 6:**
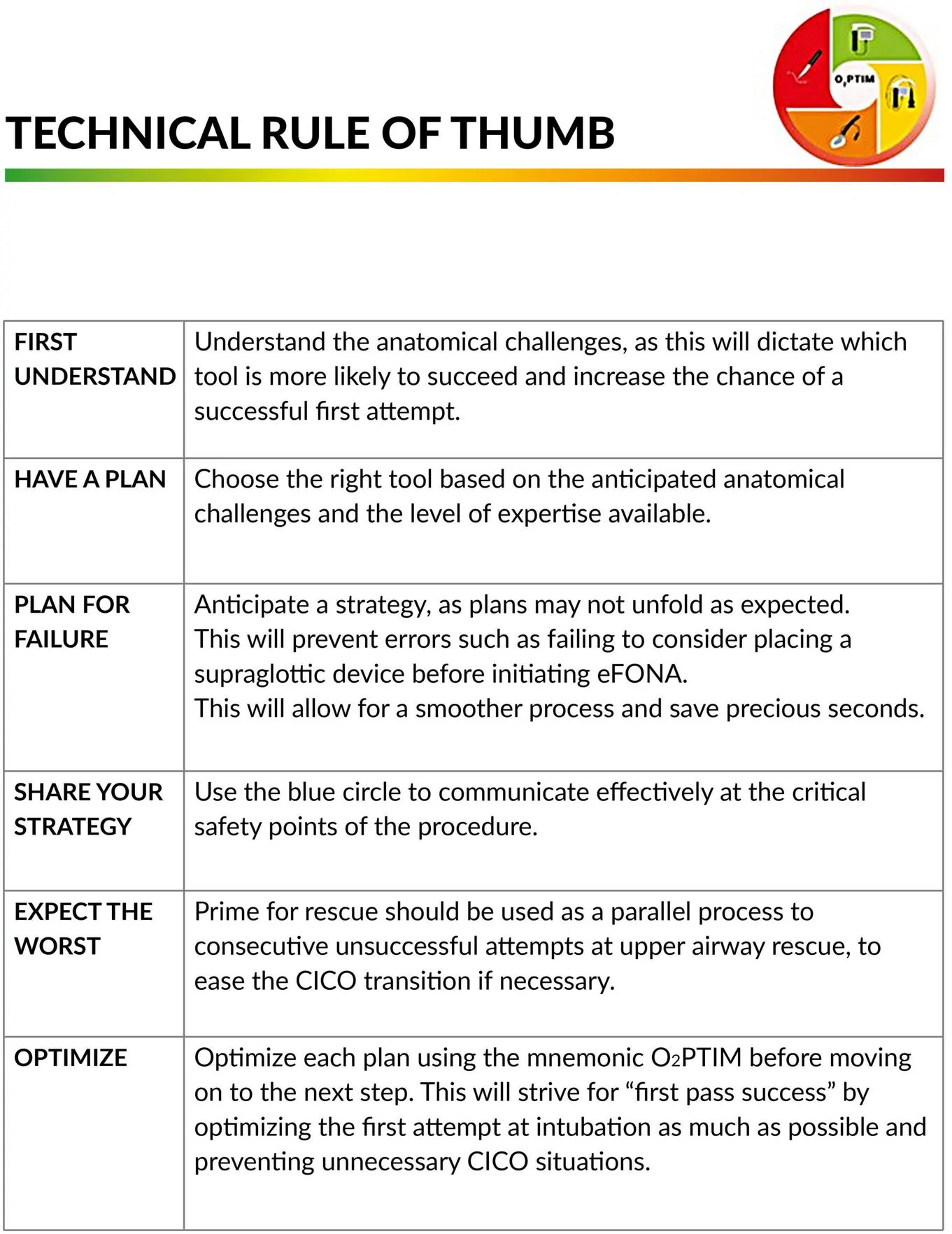
Rule of thumb for addressing the technical components of airway management: “First, understand; then plan accordingly; anticipate potential failure; communicate your strategy to the team; prepare for the worst-case scenario; and optimize performance.” FONA, front of neck access.

We propose the following pragmatic rule of thumb (55) to guide the operator through the key steps of the technical circle (see Figure 6), facilitating seamless transitions and minimizing omissions during rescue interventions while prioritizing first-pass success.

**FIGURE 7:**
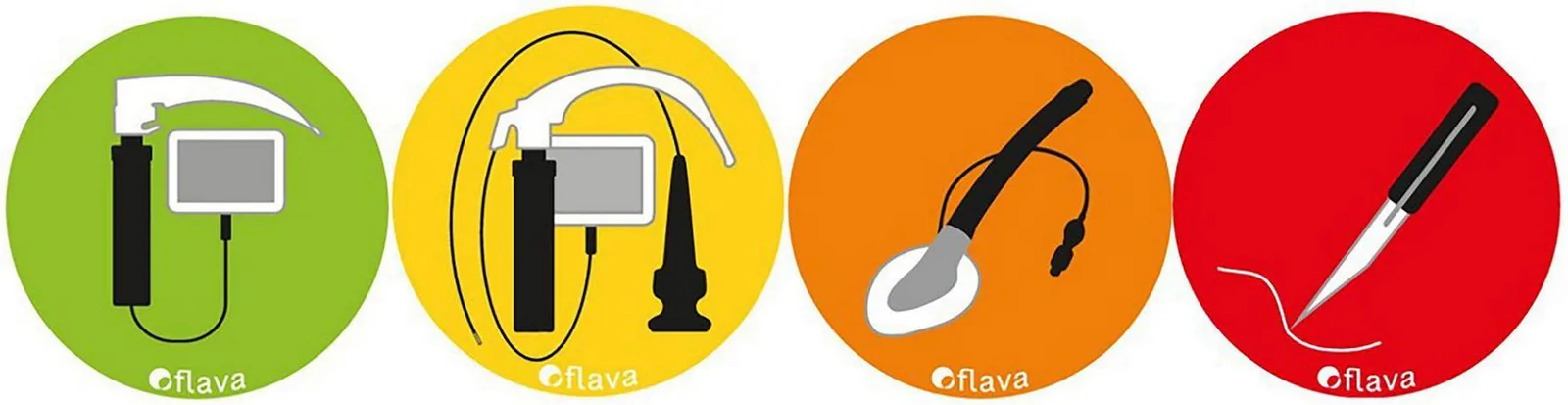
Use of distinct color coding to differentiate each management plan.

Each color denotes a specific airway management plan, with each plan providing a goal-directed cue for a tailored intervention (i.e., a specific kind of airway device) aimed at addressing a distinct anatomical challenge (Figure 7). Based on current evidence (56–59), Macintosh blade video laryngoscopy is now our first choice for the green plan, hyper angulated blade video laryngoscopy, on the other hand, is part of the yellow plan. For a detailed description of the plans, readers are referred to our previous publication (10).

**FIGURE 8:**
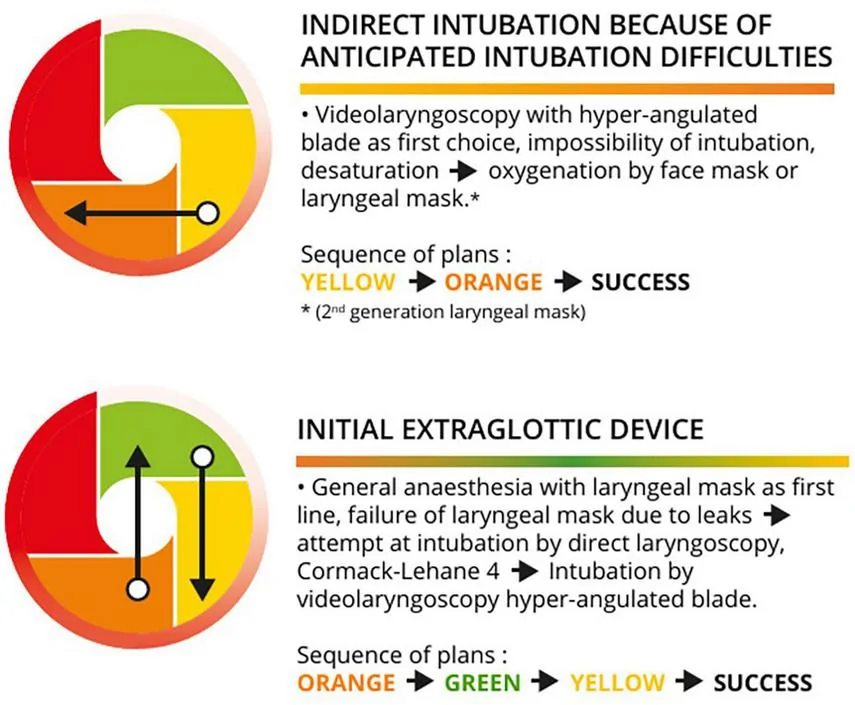
Illustrative examples of context-specific SWAGs strategies. To better reflect real-world practice, the selection and sequencing of management plans are adapted to the clinical context and guide the overall strategy; accordingly, progression does not necessarily follow a fixed green-to-red sequence. SWAGs consistently begin with a briefing phase in which the initial strategy is defined, with further refinement occurring during the SOS phase as needed.

Planning for potential failures and communicating these plans to the team is crucial for aligning expectations and facilitating coordination (43). Effective failure planning involves the development of a well-defined strategy (Figure 8). A robust strategy constitutes an optimal combination of contingency plans tailored to the specific context of the situation. Illustrative examples of such strategies are provided in our foundational study (10).

To optimize decision-making efficiency during airway management, the selection of airway devices allocated to each management plan was deliberately restricted to those most likely to overcome specific anatomical challenges. “Choice overload” has been shown to contribute to decisional delay, omission errors, and action paralysis (60). Consequently, limiting the range of immediately available airway equipment may reduce cognitive load, preserve critical time, and enhance decision quality. In accordance with the principle *of less is more* (61), a restricted selection also promotes greater operator familiarity with the available devices. Inadequate equipment availability has been identified as a contributing factor in airway-related complications (17). To mitigate this risk, airway device accessibility is facilitated through the implementation of a structured airway cart and a dedicated airway bag, described below.

Usability, defined as the extent to which a device can be employed intuitively, efficiently, and safely in high-acuity situations, is inherently influenced by device design and manufacturer-specific characteristics (3). For this reason, final device selection remains guided by user experience and preference.

**FIGURE 9:**
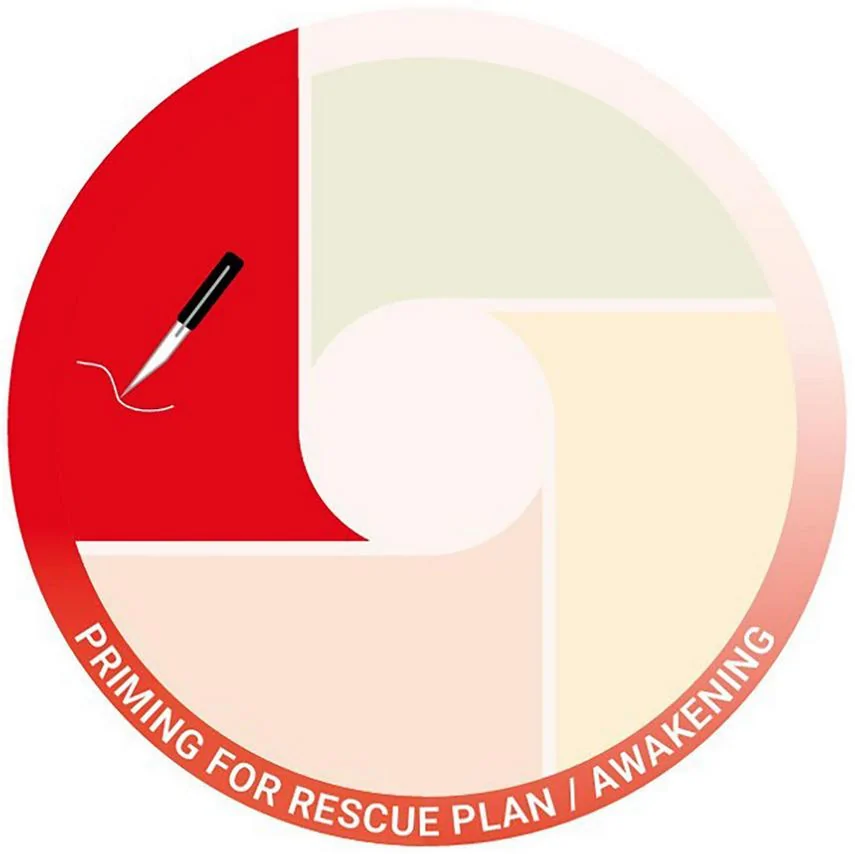
Dedicated visual cue for CICO situations. Transition to FONA is psychologically challenging. The increasing intensity of the red circle toward the red plan serves as a visual cue to prompt early consideration of CICO scenarios, thereby facilitating timely escalation to FONA.

The infrequent yet critical decision to transition to front of neck access (FONA) is supported through the integration of a dedicated visual cue linked to predefined “critical language,” prompting early consideration of a cannot intubate, cannot oxygenate (CICO) scenario (Figure 9). Critical language refers to standardized communication in which specific terms carry explicit, mutually agreed-upon meanings (62). This structured communication strategy reduces ambiguity, flattens hierarchy, and enhances shared SA within the clinical team (62).

#### 4.2.3 The non-technical circle and its mnemonic

**FIGURE 10:**
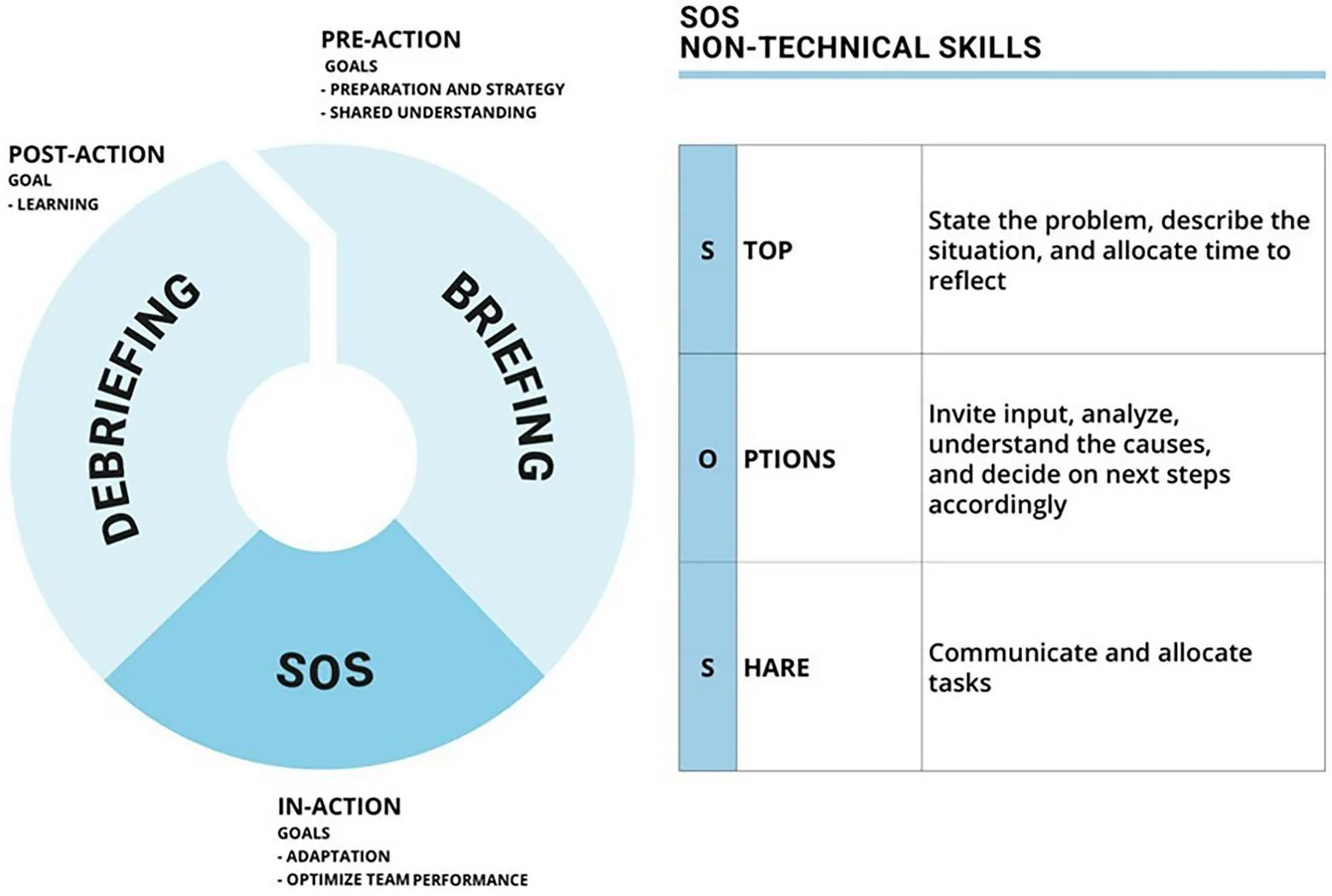
Overview of the SWAGs non-technical cognitive aids. A full description can be found elsewhere (10).

Airway management constitutes a collective competency, necessitating effective information sharing among team members to establish and sustain an accurate shared mental model, which is essential for optimal performance and favorable patient outcomes (54). The non-technical blue circle, along with its associated mnemonic (Figure 10), has been developed to facilitate NDM by promoting team reflexivity (TR). TR is a deliberate process of discussing team goals, processes, or outcomes. It can function as an antidote to team-level biases and errors in decision making (63, 64). A comprehensive description of cognitive biases in anesthesiology is beyond the scope of this article and interested readers may consult other sources (8).

TR fosters accurate mental models (65) and thus promotes SA, the heart of RPD (39, 40, 43). In the setting of NDM, effective decision making largely depends on having a good understanding of the situation at hand (13).

Experienced decision makers assess and interpret the current situation and select an appropriate course of action based on conceptual patterns stored in their long-term memory as “mental models.” Environmental cues activate these mental models, which then guide the decision-making process. The reader is referred to the original model of SA developed by Mica Endsley et al. (66) and the role of SA in NDM (13).

**FIGURE 11:**
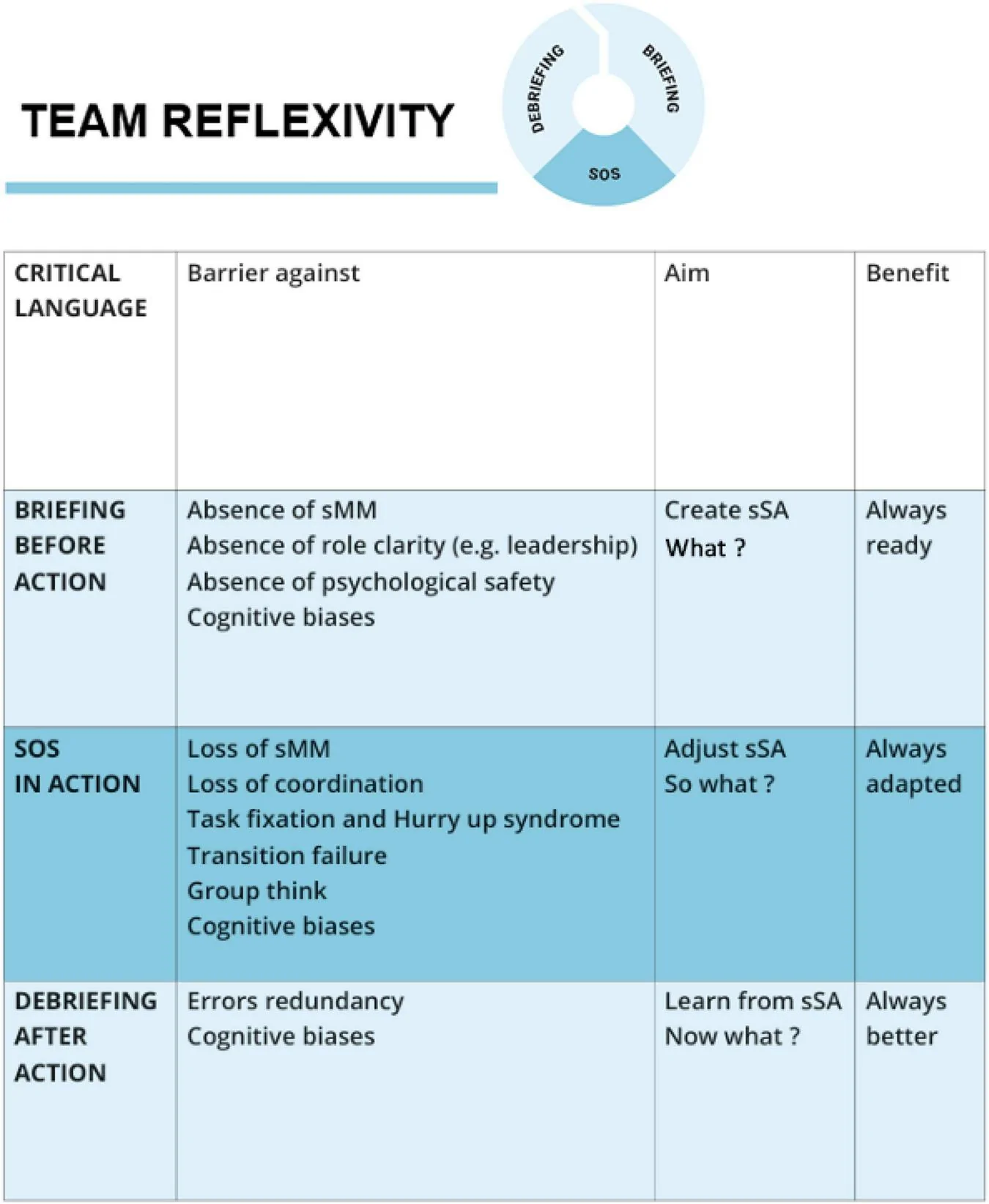
TR across three temporal phases to promote shared SA. TR triggers deliberate team-level reflections at three critical safety times, with three different objectives. sSA, shared situation awareness; sMM, shared mental model.

TR performed at three critical times (Figure 11), facilitates a dynamic evaluation of SA using the “what, so what, now what” framework (43). This iterative process supports continuous, context-sensitive decision-making during airway management, enabling the selection of the most operationally appropriate interventions (39) while proactively mitigating potential errors. The incorporation of a “sterile cockpit” approach during these structured huddles has been demonstrated to further enhance team performance by minimizing distractions and optimizing cognitive focus (67).

Briefing is recommended to precede any deployment of the SWAGs and should represent the preferred point of entry into the cognitive aid, as it allows the team to establish a shared mental model of the situation before initiating action (43). The leader must be explicitly named and the airway strategy clearly outlined, considering any anatomical or physiological challenges identified by all team members. Back-up plans must also be clarified. Finally, roles should be clearly assigned to define areas of responsibility. The leader should actively promote psychological safety to flatten the hierarchy and facilitate open communication (e.g., encouraging people to speak up) (26, 68).

The mnemonic “SOS” is used during the action. It prompts re-evaluation when workflow or shared mental models are disrupted, and mitigates individual or team fixation, such as excessive effort on failing techniques (1), or susceptibility to groupthink. Groupthink occurs when the desire for group harmony or conformity overrides the realistic appraisal of alternatives, leading to flawed decisions. The team bends the facts (they are ignored, rejected or argued away) to fit disconfirming cues (43). Additionally, it facilitates transitions between plans and supports coordinated workflow. Functionally, it serves as a cognitive “handbrake,” mitigating the tendency toward “hurry-up syndrome” (44), and operates as a cognitive forcing strategy (69). This concept was described by Rall et al. as the 10 s-for-10 min principle (47). Furthermore, it may reduce barriers to communication by encouraging team members to contribute supplementary information (68), thereby minimizing the risk of actions based on an inaccurate or incomplete mental model.

Debriefing should also be systematically conducted, following the completion of an airway intervention and may encompass immediate defusing, structured feedback, or a comprehensive debriefing session, according to team needs and contextual constraints. Importantly, debriefing should extend beyond the traditional Safety I approach, which focuses primarily on the identification and mitigation of errors or adverse events, to also incorporate a Safety II perspective that examines and reinforces successful performance (70). This dual approach enables a more comprehensive understanding of airway management safety. Such reflective practice supports continuous professional development, strengthens shared mental models within the team, and contributes to the consolidation of collective memory, thereby shaping and sustaining the local culture of airway management.

#### 4.2.4 The airway cart and the airway bag

**FIGURE 12:**
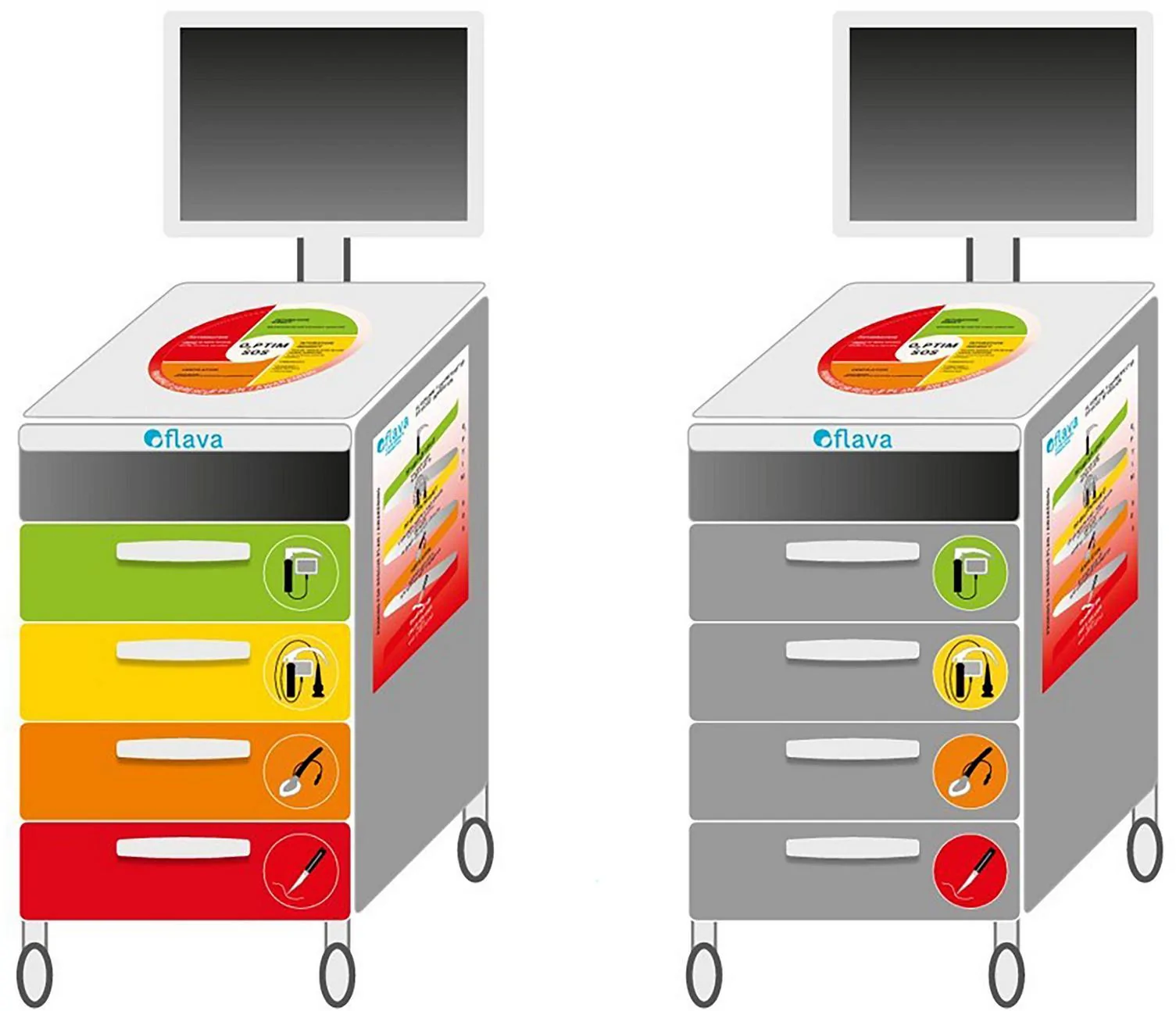
The SWAGs airway cart.

The design of the SWAGs airway cart (Figure 12) represents a critical component of the clinical environment in airway management (49, 71). When optimally organized, it provides the most beneficial support intervention for decision-making (15), by ensuring immediate access to the devices required to implement the strategies delineated by the two-circle framework. Consequently, the airway cart and associated cognitive aids function as interdependent and fully aligned elements of the system. From a systems thinking perspective (72), misalignment among interdependent parts of a system may compromise both, quality and safety (49).

A color-coded icon system establishes clear visual correspondence between the two circles and the cart drawers, effectively extending the cognitive framework into the physical workspace. Lomax et al. (49) have shown that standardized color coding and compartmentalization enhance visual search efficiency, facilitate error detection, and support metacognitive processes, thereby introducing an additional layer of safety in anesthetic procedures. This organization frees attentional resources, allowing clinicians to focus more effectively on task performance.

Each drawer contains the necessary devices for its corresponding plan, adhering to the “less is more” principle (61). Only equipment required to address specific anatomical challenges is prioritized, thereby preventing choice overload and reducing the number of techniques that clinicians must master (71). The use of a CICO kit is preferable, as it enables rapid deployment without the need to recall or assemble individual components during time-critical emergencies (71).

Timely access, a known barrier to airway management success (17), is crucial for a successful implementation and acts as powerful cognitive cues influencing decision-making (3). This depends on strategic placement and mobility of the cart. Lastly, regular elective usage of the airway cart must be encouraged in clinical practice to fully encompass its content, emphasizing the name of “airway cart” instead of “difficult airway cart”.

**FIGURE 13:**
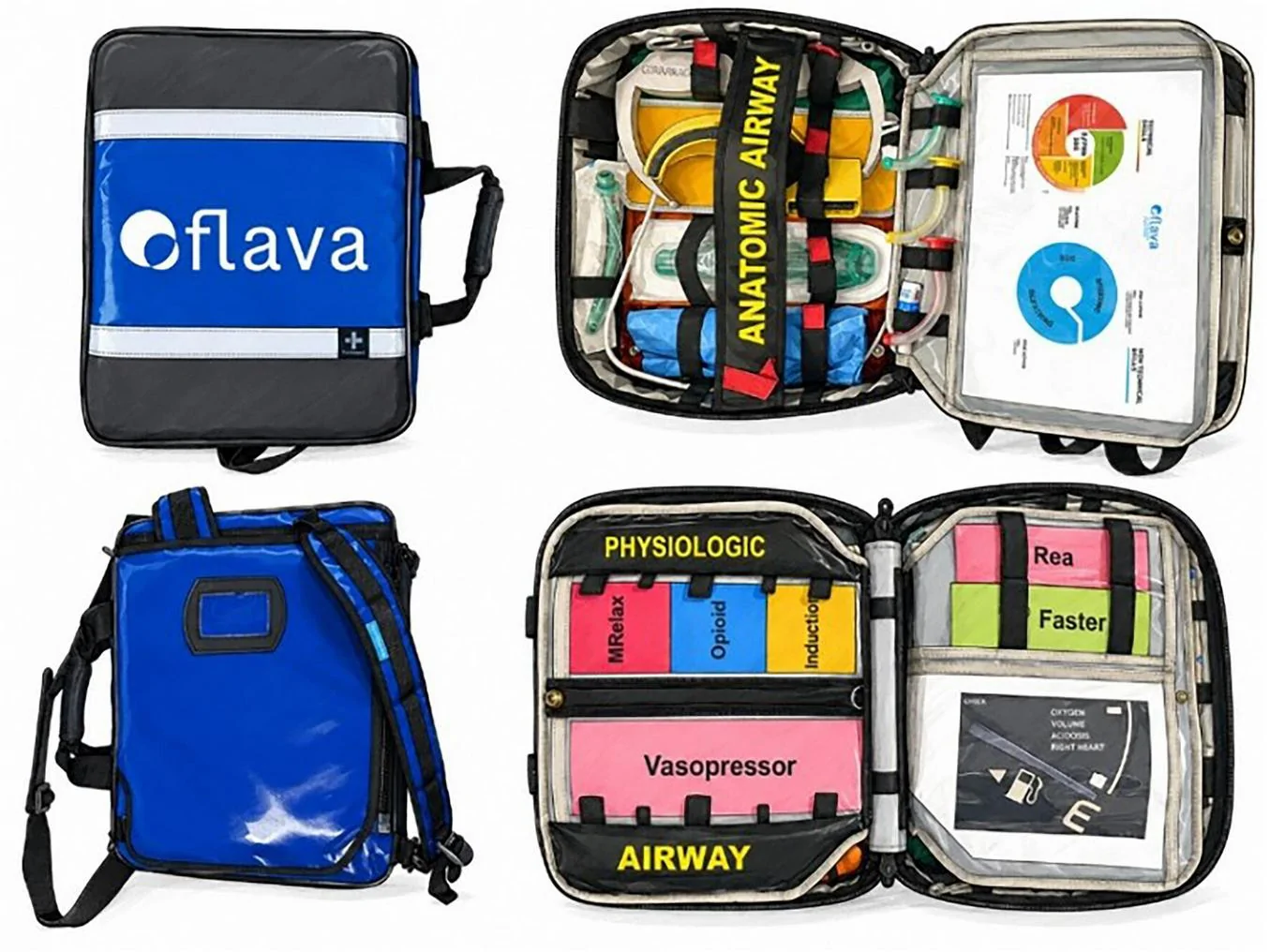
The SWAGs airway bag.

FLAVA also developed a streamlined SWAGs airway bag designed (see Figure 13) for use in restricted-access settings while also reducing the financial burden associated with fully equipped airway carts. Moreover, this increased visibility is anticipated to enhance familiarity and SA of the SWAGs, supporting effective implementation.

### 4.3 Designing environment and organization

Failure to account for the integration of cognitive aids within the broader work system may predispose healthcare professionals to suboptimal performance (73). Although survey studies have reported generally positive attitudes toward cognitive aids (around 80%), actual routine use has been considerably lower (approximately 7% in one study) (74, 75), highlighting the gap between acceptance and implementation. These findings may not be generalizable to all healthcare systems. The absence of a supportive clinical and educational culture has been identified as a major barrier to effective implementation (11, 51). Airway management outcomes are shaped by multiple interacting factors operating at individual, team, organizational, and environmental levels (16). These interdependencies underscore the necessity of a systems-based approach to the design and deployment of decision-support tools. Achieving alignment across these domains is essential to ensure that cognitive aids are not only theoretically sound but also intuitive, resilient, and sustainably embedded in routine clinical practice.

#### 4.3.1 Integrating SWAGs into a patient journey

**FIGURE 14:**
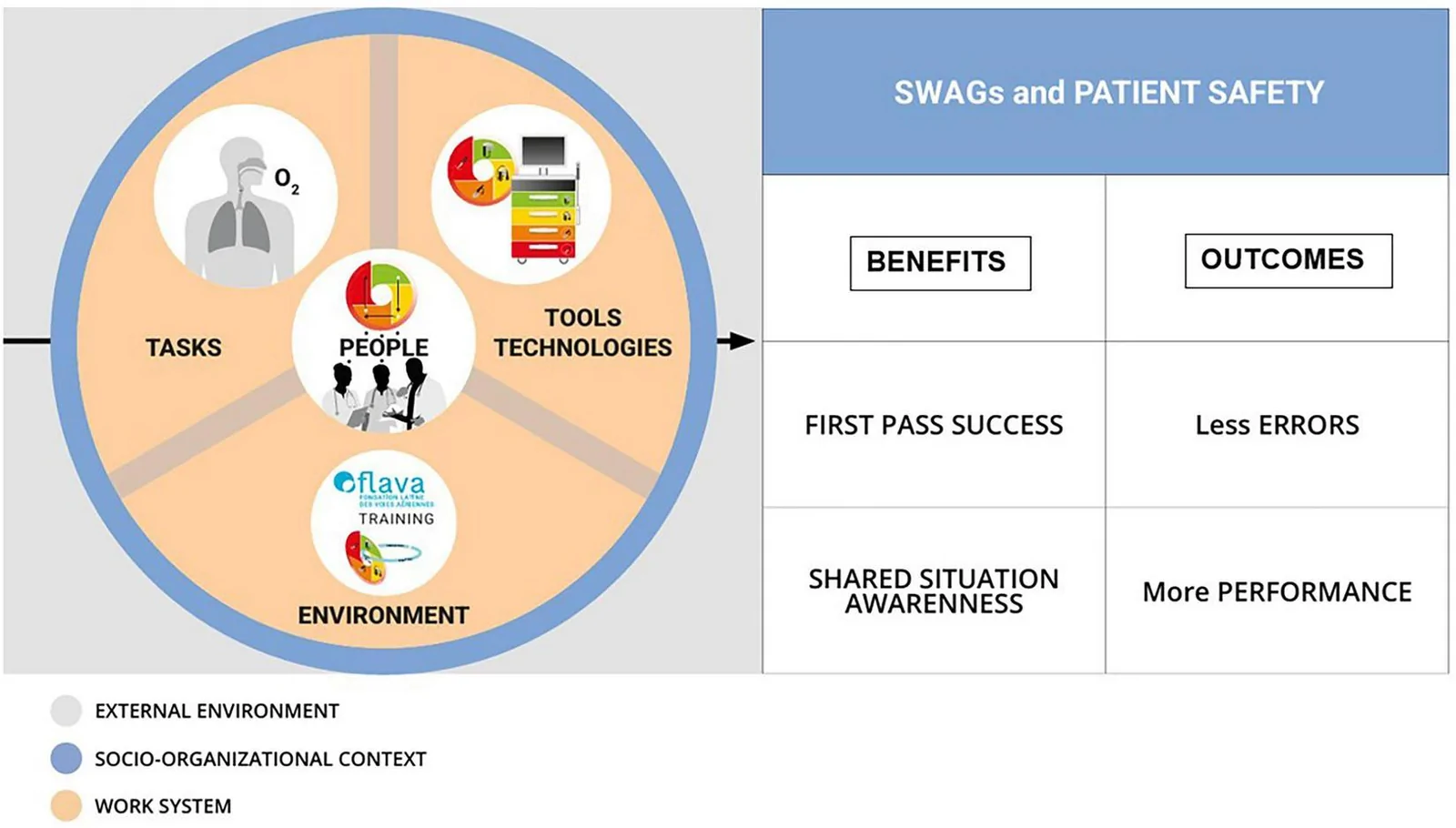
SWAGs airway management work system represented as a sociotechnical model, adapted and modified from SEIPS 101 framework (19) to integrate the SEIPS 3.0 Model (18).

The application of the SEIPS 3.0 (18) and 101 models (19) enhances both theoretical and methodological rigor by providing a structured framework through which airway management can be conceptualized as an integrated work system (Figure 14).

Patients may require airway management at some point to undergo surgery, to treat respiratory failure, or to secure the airway in the event of cardiac arrest.

To successfully oxygenate the lungs (task), we need a combination of technical skills, i.e., using the right tool for the right anatomical challenges (tools and technology: the four-color circle and the airway cart), and social and cognitive skills (non-technical skills), i.e., creating, adjusting and learning from sSA to support decision-making (tools and technology: the blue circle). These principles are illustrated in Figure 14. Tools and technology must be designed with an understanding of how experts make decisions under pressure (people). Training in this holistic approach is crucial to ensure successful implementation (environment). The outcomes (increased sSA and performance, decreased cognitive load and errors) are tailored to the airway provider and serves to improve safety in airway management.

#### 4.3.2 Promoting efficient implementation

**FIGURE 15:**
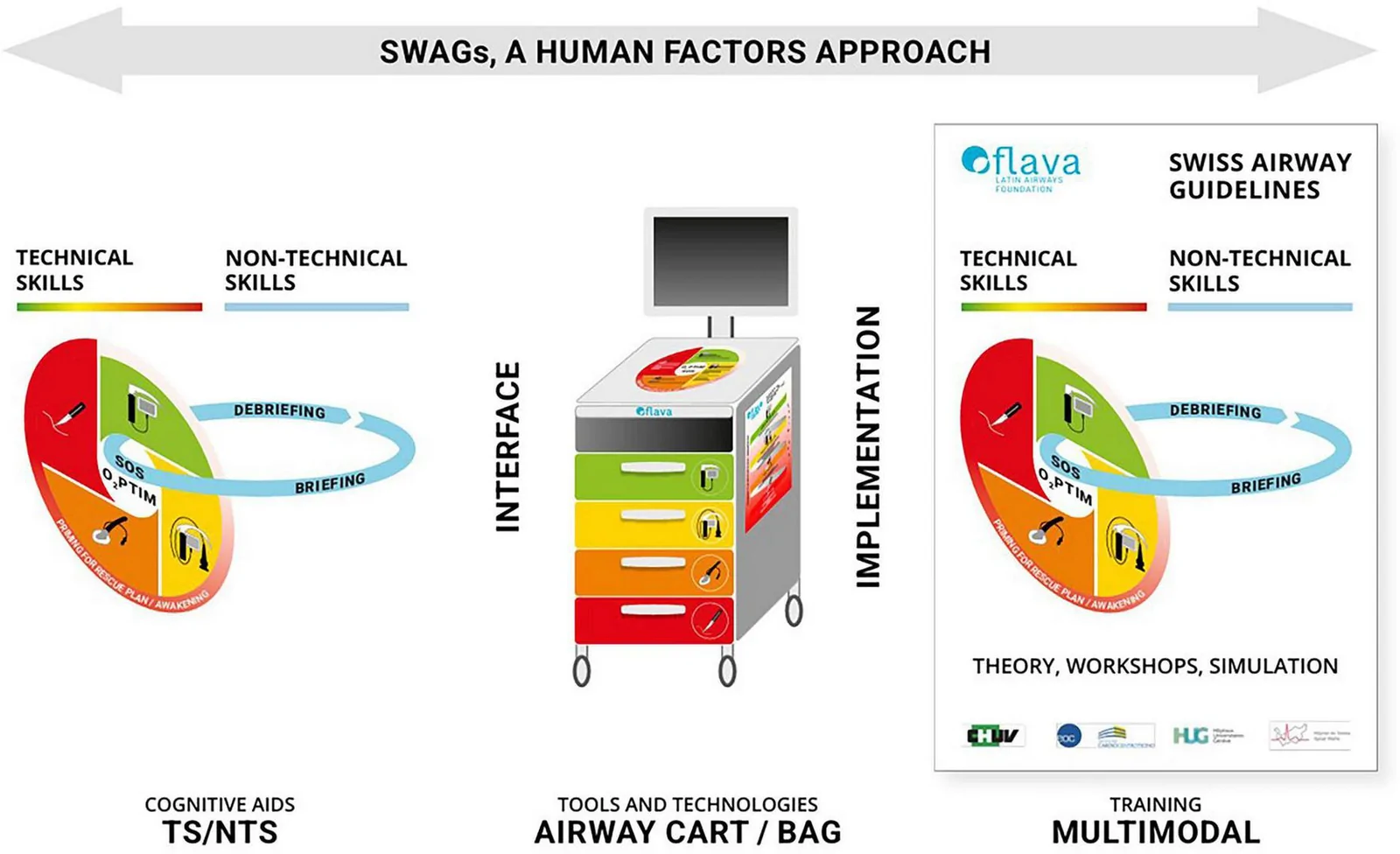
Holistic framework underpinning the SWAGs approach. Taking a holistic approach to consider and align all parts of the work system (tasks, people, tools, technologies and training) is likely to improve implementation and ultimately promote patient safety and user wellbeing.

Alignment of cognitive aids, airway cart, and training under a shared framework is linked to improved team coherence and crisis response (3) (Figure 15).

HFE principles are increasingly embedded in contemporary difficult airway guidelines (1, 76). Across these guidelines, safety is operationalized through structured cognitive aids, early escalation pathways, and an emphasis on non-technical skills and team-based crisis management, reflecting a shift from purely algorithmic approaches towards system-aware and adaptive models of airway care. This integration in the SWAGs is summarized in Table 2, which depicts the alignment between HFE principles and the SWAGs.

**TABLE 2:** Summary of the integration of HFE in the SWAGs.

Human factors contributions to SWAGs conception	Human factors domains	Application of human factors in SWAGs
Understanding the task *VUCA* *Complex Adaptive System* *Resilience Engineering*	Task and work analysis	Task context: ° Why is airway management challenging? Task nature: ° How does performance emerge from the system? Task solution: ° How is performance maintained despite variability and uncertainty?
Understanding the people *Situation Awareness* *Naturalistic Decision-making* *Cognitive load theory* *Information processing theory*	Cognitive human factors	Optimization of human performance under uncertainty: ° human capabilities ° human limitations ° adaptive decision-making
Designing tools and technologies *User-centered design* *Situation Awareness design* *Team reflexivity*	Decision-support design	Reduction of cognitive load: ° cognitive aid → SWAGs cognitive aids ° visual prompts → color-coded and icon coded system ° anticipation, adaptation and in-action learning using structured communication → NTS circle, Briefing, SOS, Debriefing Enhancement of situation awareness: ° team reflexivity → NTS circle ° simplified visual prompts aligned with decision-making process Optimization of first-pass success: ° cognitive aid, equipment standardization, availability, and usability → TS circle, O2PTM, airway cart and airway bag ° streamlined technical principles → color and icon-coded system, less is more, integration of choice overload risk Context-sensitive decision support: ° cognitive aid → SWAGs cognitive aids ° recognition-primed cues → color and icon coded system Adaptive and flexible decision support: ° circular approach in line with the work-as-done
Implementing within the system *SEIPS models* *Systems design* *Implementation science*	Human-centered systems design	System integration and implementation: ° Alignment of tasks, workforce, technologies, and care environments ° Multimodal and interprofessional training approach ° Integration across the patient care pathway ° Harmonized implementation across Swiss Hospitals
Overall goal Effective decision-support systems should fit human cognitive, social, and operational capabilities rather than requiring humans to adapt to system limitations.		

Standardization across hospitals within our regional network, promotes consistency and its adoption among rotating fellows in anesthesia (10). We furthermore advocate identification of institutional airway leads in every hospital to ensure smooth local deployment, as suggested by airway societies (77).

Training using a blended approach (scientific lectures, hands-on workshops and high-fidelity simulation) ensures opportunities to learn about human factors principles, update evidence-based practice, use tools and technologies, practice non-technical skills under pressure and test the usability of the SWAGs. Ongoing feedback from users enables the necessary adaptations to be made to overcome the challenges at the sharp end. According to the “hierarchy of control model” (22), training is an essential part of ensuring smooth adoption. However, it does not compensate for poor system design (21, 22, 73).

## 5 Strength and limitations

This approach, promoting vigilance, favoring flexibility over rigid rules, and designing systems that support rather than constrain operators, mirrors the strategies of High Reliability Organizations, which sustain safe performance despite inherently high-risk work (78).

The SWAGs were informed by human factors engineering, cognitive psychology, and the airway management literature, but remain a design framework rather than a validated clinical intervention. Restricting device choice may enhance cognitive efficiency under crisis conditions yet could also oversimplify complex scenarios or fail to account for local expertise, equipment availability, or patient-specific factors. Clinicians generally view cognitive aids favorably, but routine use remains limited (74, 75), implementation depends on more than design quality alone. Organizational culture, multidisciplinary simulation training, and sustained institutional support are likely to determine successful adoption (11, 51). Because the SWAGs were developed within the Swiss airway management network, their transferability to other healthcare systems requires local adaptation and prospective validation rather than assumption.

As a narrative review, this work does not follow a formal systematic review methodology, and selection of evidence may therefore be subject to selection bias.

Further work should combine qualitative approaches, clinician experience, team dynamics, contextual factors, with quantitative evaluation of safety outcomes and implementation impact, to strengthen the evidence base and guide scalable improvements in airway management systems.

## 6 Conclusion

We describe the SWAGs, an evidence-informed cognitive aid now ready for validation. Grounded in HFE and informed by the SEIPS models, they embody a system-design approach that optimizes human performance while leveraging clinical expertise. Given the complexity and high-stakes nature of airway management, system design must align with human cognitive and adaptive capabilities; poorly designed systems constrain performance and increase the risk of failure.

Developed by the FLAVA as a series of implementation tools, the SWAGs support clinicians by simplifying decision-making and promoting sSA during difficult and routine airway management. Their structured, ergonomically informed interface uses intuitive, color-coded visual prompts to enhance both social and cognitive skills (TR, SA) and to highlight goal-directed cues critical for technical performance. By strengthening both domains, the SWAGs are intended to foster sSA and improve first-pass success. Based on current evidence from human factors and cognitive aid research, these design features are expected to facilitate team performance; their impact on usability, implementation, clinician performance, and patient outcomes remains to be established through prospective simulation and clinical studies.

More broadly, the SWAGs promote a holistic, system-based approach that aligns tasks, individuals, tools, and technologies, making safe practice the path of least resistance. This work extends the HFE reframing proposed in our introduction from theory to practice, demonstrating that reliable airway safety is achieved not by demanding more from individuals, but by deliberately designing systems that support, rather than hinder, human performance.
